# Supplementary material for: The Par3 polarity protein is an exocyst receptor essential for mammary cell survival
Source: Nat Commun. 2017 Mar 30;8:14867. doi: 10.1038/ncomms14867 (PMC5379108; doi:10.1038/ncomms14867)
Supplement: Supplementary Information — Supplementary Figures. [file ncomms14867-s1.pdf]

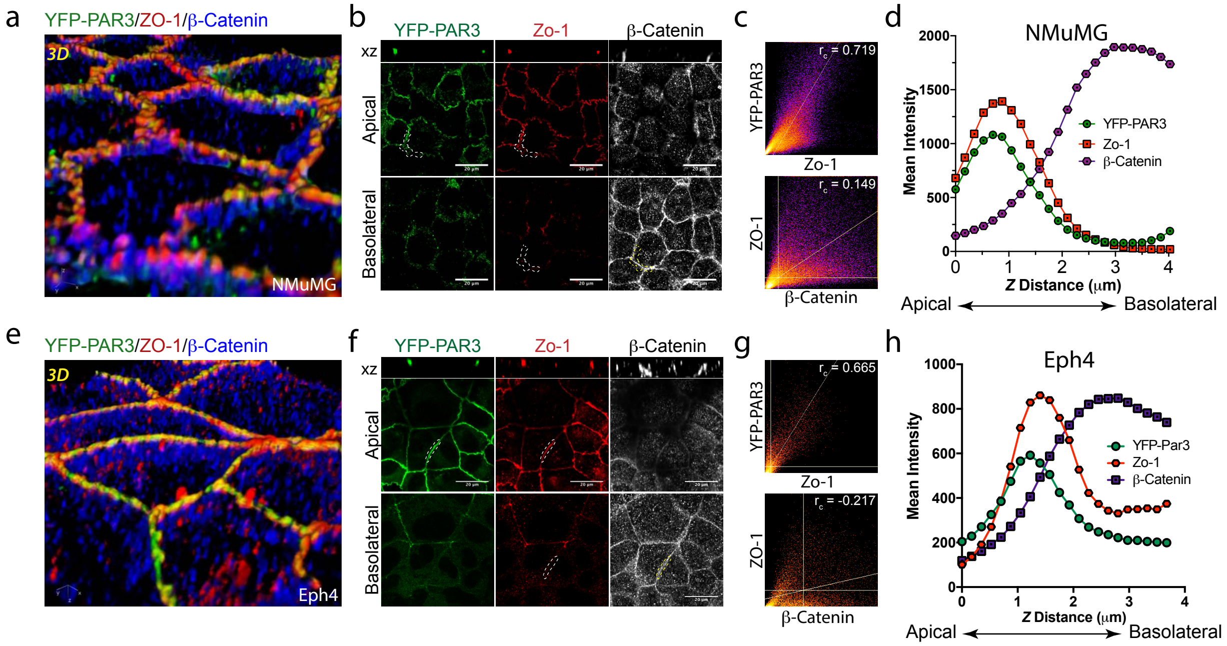

Supplementary Figure 1. Apical and lateral proteins are correctly localized in NMuMG and Eph4 cells. NMuMG and Eph4 cells stably expressing YFP-PAR3 were allowed to form a confluent monolayer, fixed and immunostained for endogenous Zo-1 and β-catenin. **(a)** 3D rendering of confocal z-stack images of NMuMG cells deconvoluted using the 3D-blind deconvolution algorithm. **(b)** Selected slices from apical and basolateral regions shown in **a**. **(c)** Quantification of colocalization between YFP-Par3 and Zo-1, or Zo-1 and β-catenin from the ROIs shown in the images in **b** using the Pearson correlation coefficient. r_C = pearson coeffient of colocalization. **(d)** Z-intensity profile of YFP-PAR3, Zo-1 and β-catenin through 4μm Z-slices. (**e-h**) Image analysis as in **a-d**, using Eph4 cells.

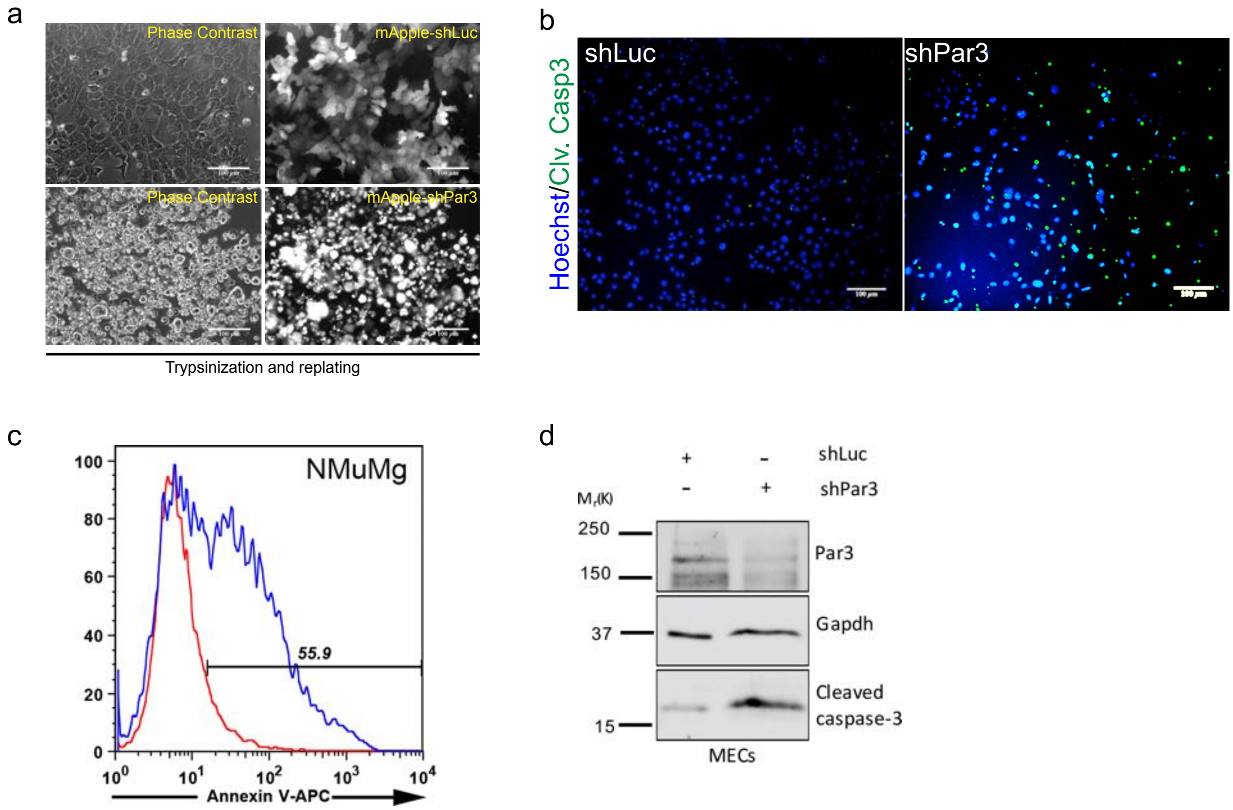

Supplementary Figure 2. Loss of Par3 induces apoptosis. Related to Figure 1. **(a)** Knockdown of Par3 in NMuMG cells prevents cells from re-attaching on to the plate. NMuMG cells were transduced with lentivirus expressing mApple together with either shLuc or shPar3. Two days after transduction cells were trypinized and re-plated and images taken on day 3. Scale bar, 100μm. **(b)** Cleaved Caspase-3 and Hoechst 33342 staining in NMuMG cells transduced with shLuc or shPar3. Scale bar,100μm. **(c)** Flow cytometry of Annexin V stained NMuMG cells transduced with shLuc or shPar3 containing lentivirus. Red = shLuc, Blue = shPar3. **(d)** Immunoblot of Par3, Gapdh and Cleaved Caspase-3 in primary mammary epithelial cells transduced with lentivirus containing shLuc or shPar3. All experiments were successfully repeated at least two times.

a

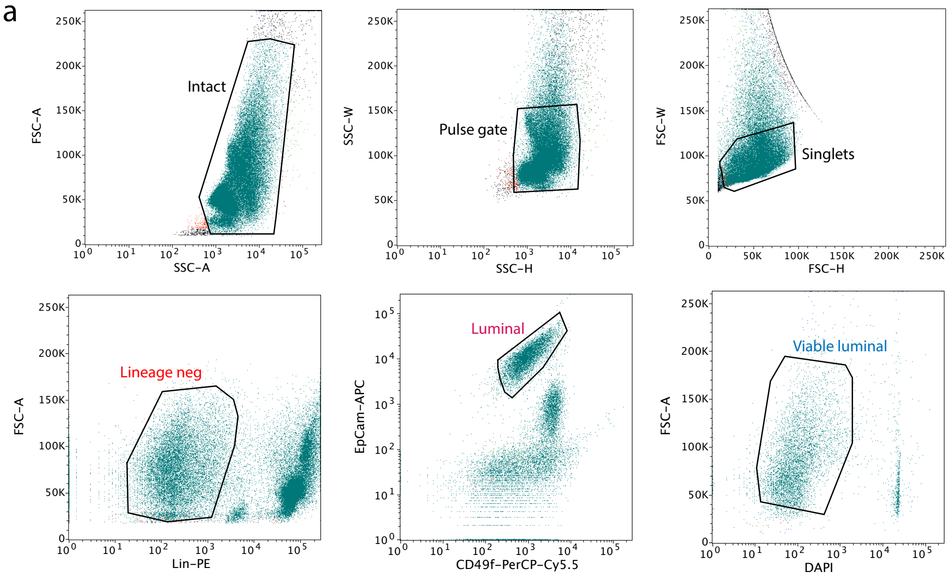

b

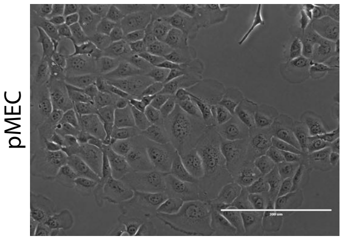

Supplementary Figure 3. Fluorescence activated cell sorting of primary luminal mammary epithelial cells from C3H mouse. Related to Figure 2. **(a)** Viable (DAPI-negative), CD45, CD31 and Ter119 negative mammary epithelial (lineage negative) cells expressing the EpCAMhigh/ CD49fmed markers (luminal) were isolated by flow cytometry using the appropriate lasers for the indicated fluorescent dyes. **(b)** Purified primary MECs were cultured on a laminin-coated 1 μg/ml) surface for further experiments. Experiments were repeated 3 times.

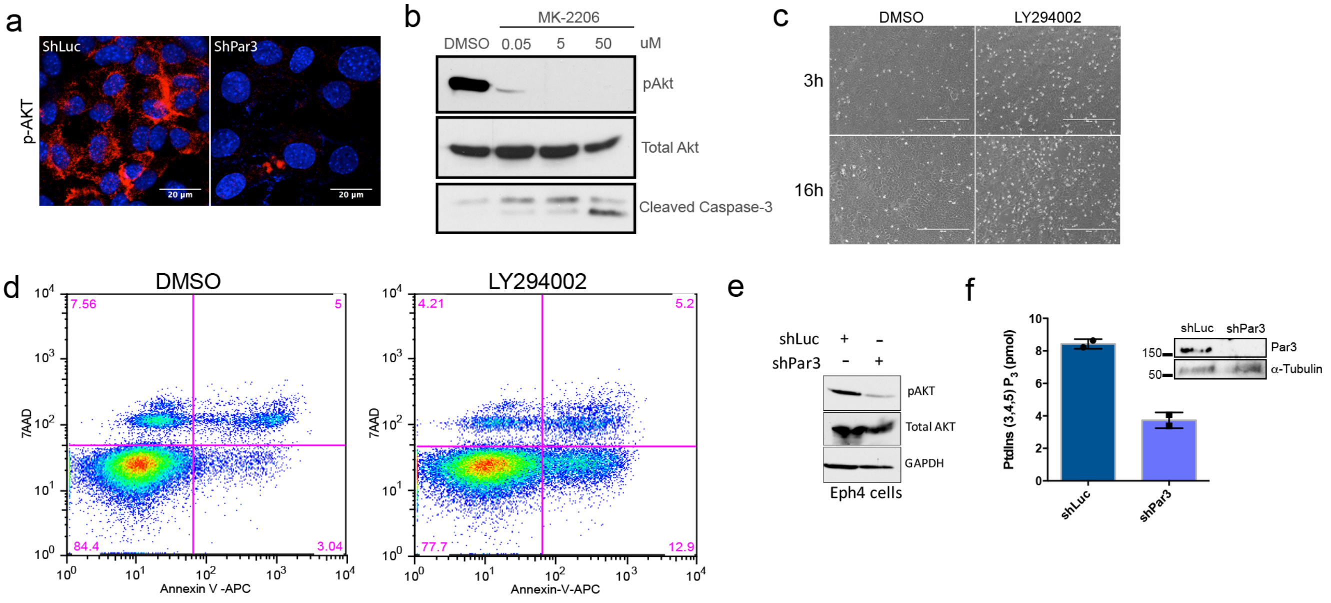

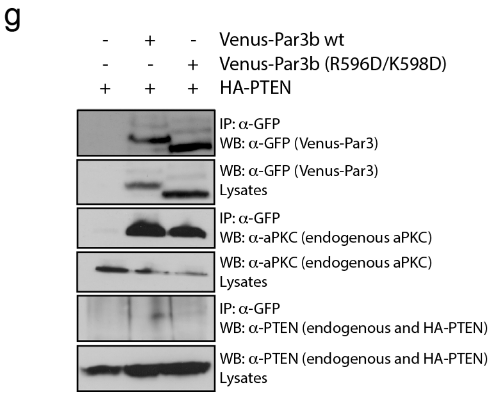

Supplementary Figure 4. Loss of pAKT in mammary epithelial cells causes apoptosis. Related to Figures 2 and 3. **(a)** pAkt staining in NMuMG cells treated with shLuc or shPar3. Scale bar, 20µm. **(b)** Immunoblot shows levels of pAkt, Total Akt and Cleaved Caspase-3. NMuMG cells treated with different concentrations of the Akt inhibitor, MK-2206, as indicated for 20h. **(c)** Phase contrast image showing NMuMG cells treated with DMSO or LY294002 (50µM) for 3h or 16h. **(d)** Flow cytometry analysis of NMuMG cells stained with 7AAD and Annexin V upon treatment with DMSO or LY294002 (50µM) for 3h. **(e)** Immunoblot showing pAkt, total Akt and Gapdh levels in Eph4 cells treated with shLuc or shPar3. **(f)** Quantification of PtdIns(3,4,5)P3 by mass ELISA. Inset shows knockdown levels of Par3 in the same pool of cells assayed. **(g)** Co-immunoprecipitation experiment and western blot analysis showing YFP-PAR3wt or YFP-PAR3b (R596D/K598D) interaction with endogenous aPKC and HA-tagged-PTEN from HEK293T cell lysates. Experiments were successfully repeated at least two times.

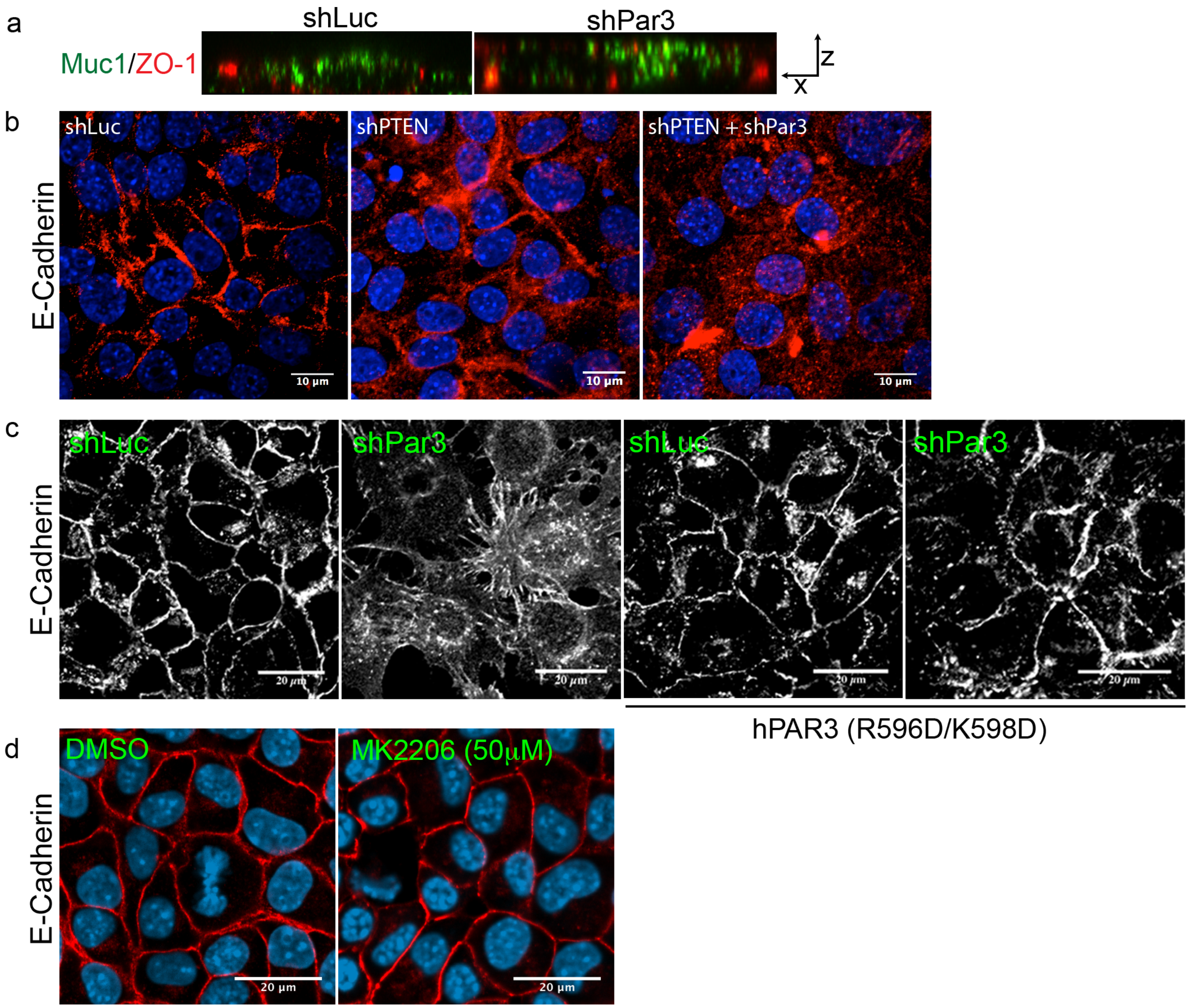

Supplementary Figure 5. Localization of Muc1 and E-Cadherin upon Par3 knockdown. Related to Figure 4. **(a)** Localization of Muc-1 (green) and ZO-1 in NMuMG cells transducted with either shLuc or shPar3 lentiviruses. Projection shows X-Z co-ordinates of a z-stack. Representative images from n=2. **(b)** E-Cadherin (red) and Hoechst 33342 (blue) staining in NMUMG cells transduced with lentivirus containing shLuc, shPten or shPar3 and shPten together. Scale bar, 10µm. Representative image from n=3. **(c)** E-Cadherin staining in naïve or YFP-hPar3 (R596D/K598D) expressing NMuMG cells that were transduced with lentivirus containing either shLuc or shPar3. Scale bar, 20µm. Representative images from n=3. **(d)** NMuMG cells treated with DMSO or MK2206 (50µM) for 7h and stained for E-Cadherin. Scale bar, 20µm. Representative images from n=2.

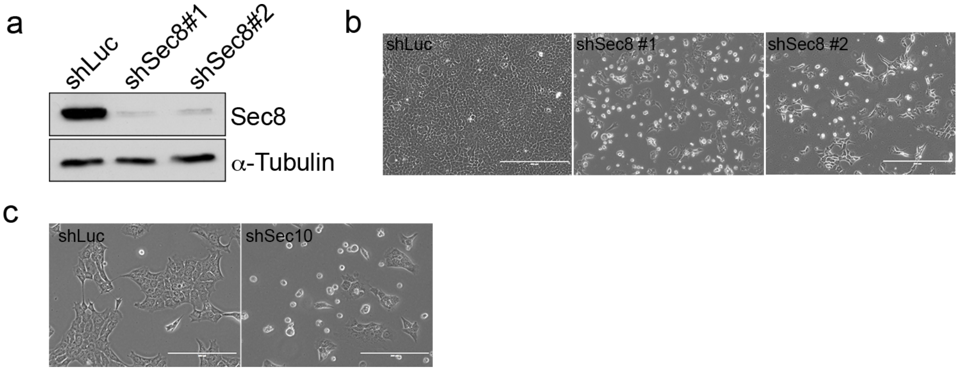

Supplementary Figure 6. Knockdown of exocyst components Sec8 or Sec10 induces cell death in NMuMG cells. Related to Figure 6. **(a)** Western blot showing knockdown efficiencies of two different hairpin RNAs targeting murine Sec8. β-tubulin immunoblot represents loading control. **(b)** Phase contrast images of NMuMG cells 3d after transduction with lentiviruses containing shLuc, shSec8#1, or shSec8#2. Scale bar, 200μm. (c) Phase contrast images of NMuMG cells 3d after transduction with lentiviruses containing shLuc, or shSec10. Scale bar, 100μm. Representative images from n=3.

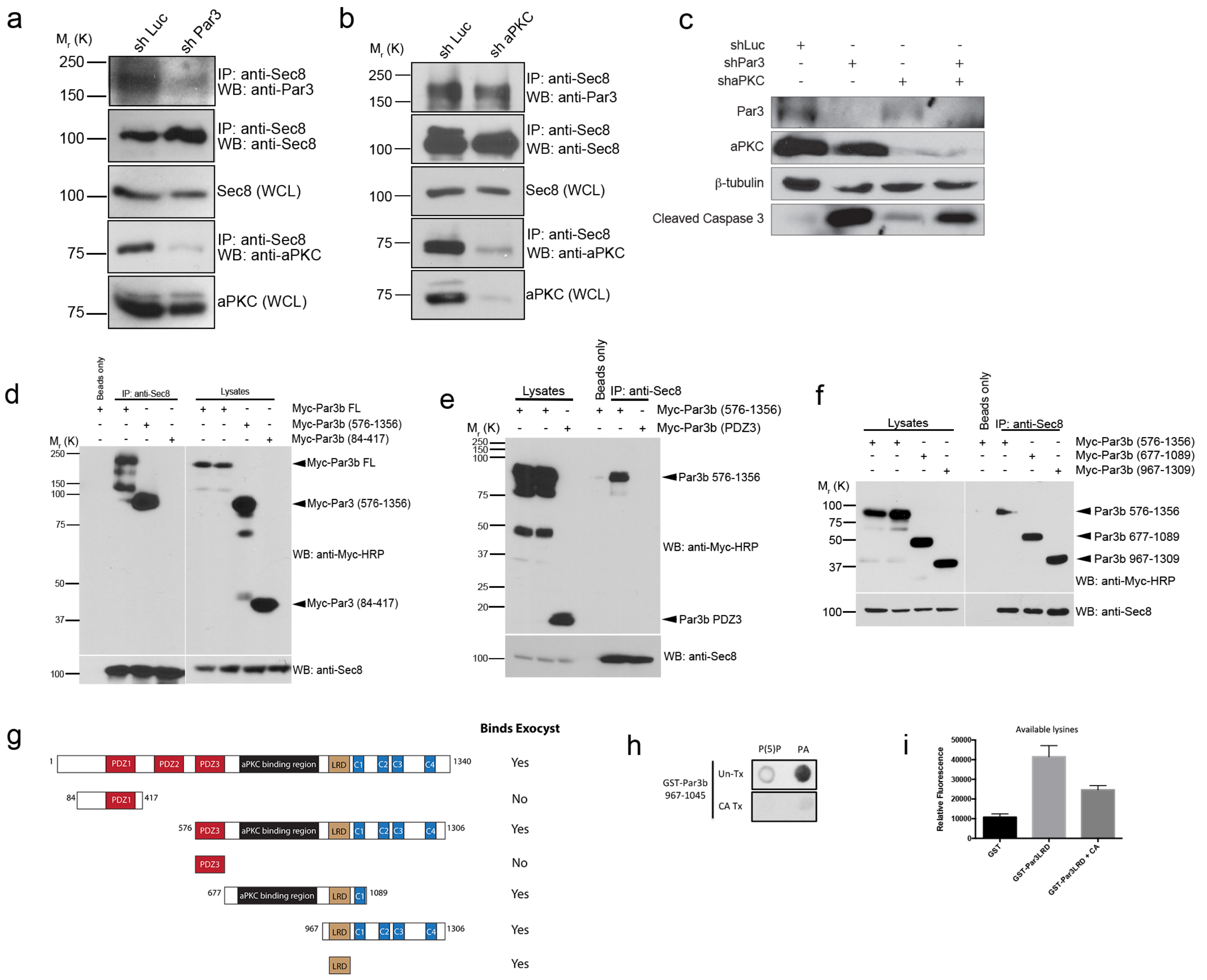

Supplementary Figure 7. Mode of Exocyst-Par3 interaction. Related to Figure 7. **(a)** Co-immunoprecipitation followed by immunoblot in NMuMG cells expressing shLuc or shPar3. NMuMG cell lysates were subjected to endogenous Sec8 immunoprecipitation with anti-Sec8 antibody. The immunoprecipates were then run on SDS-PAGE and immunoblotted with anti-Par3, anti-Sec8 and anti-aPKC to detect respective endogenous proteins. **(b)** Co-immunoprecipitation followed by immunoblot in NMuMG cells expressing shLuc or sh aPKC. NMuMG cell lysates were subjected to endogenous Sec8 immunoprecipitation with anti-Sec8 antibody followed by immunoblotting with anti-Par3, anti-Sec8 and anti-aPKC to detect respective endogenous proteins. **(c)** NMuMG cells were transduced with lentivirus containing shLuc, shPar3, sh aPKC or shPar3 together with sh aPKC. Cells extracts were collected 3d post-transduction and subjected to immunoblotting with anti-Par3, anti-aPKC, β-tubulin and cleaved caspase-3. **(d-f)** myc-tagged-Par3b full-length or different truncation mutants as indicated were expressed in HEK293T cells. Cell lysates were then subjected to immunoprecipitation using anti-Sec8 antibody and immunobloted for endogenous SEC8 using anti-Sec8 antibody. Myc-PAR3 and its mutants were detected using anti-Myc antibodies conjugated to HRP. **(g)** Schematic showing a summary of the regions of PAR3 that interact with SEC8. **(h)** Purified GST-PAR3b (967-1045), treated or untreated with citraconic anhydride (CA), binding to phosphotidylinoside (5)-phosphate or phosphatidic acid on membranes. **(i)** Available lysines upon CA treatment was assessed using ortho-phthalaldehyde fluorometric assay.

Supplementary Figure 8

Figure 1a

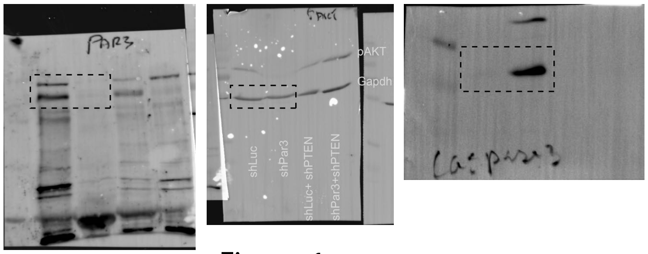

Figure 1b

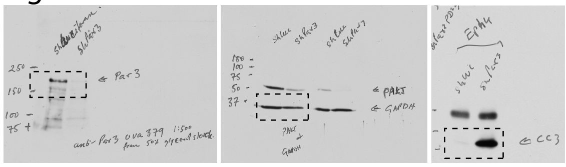

Figure 2a

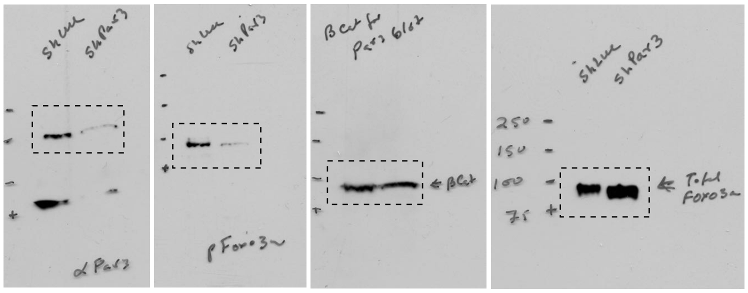

Figure 1c

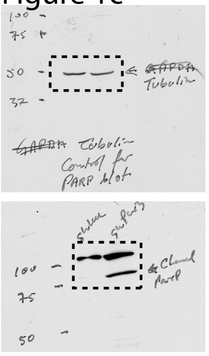

Figure 1g

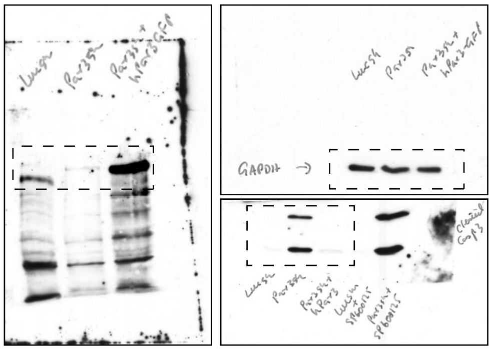

Figure 2h

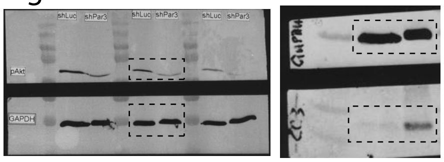

Figure 2b

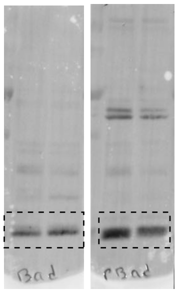

Figure 2c

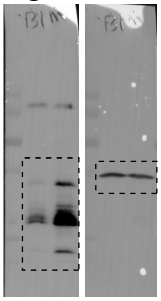

Figure 2d

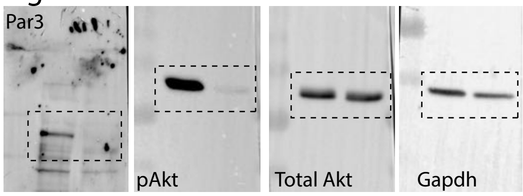

Figure 2j

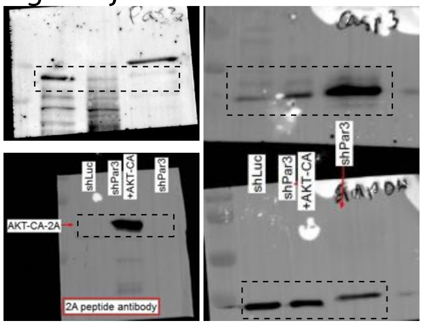

Figure 3a

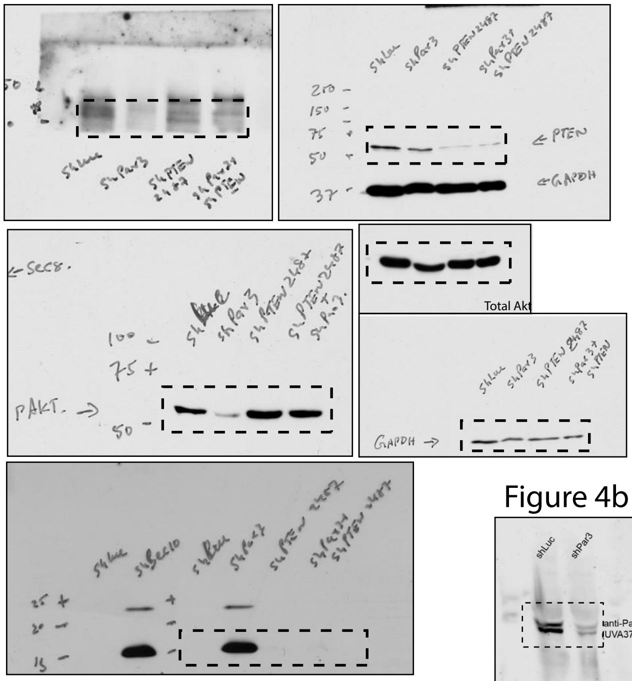

Figure 3b

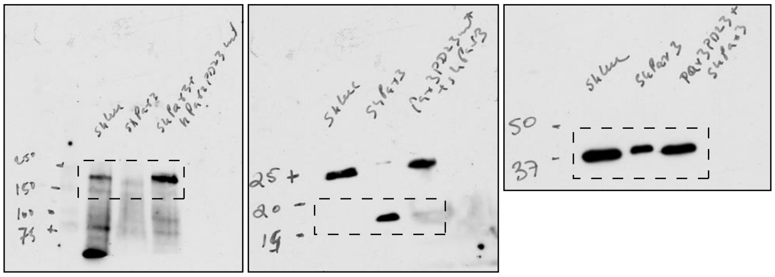

Figure 4b

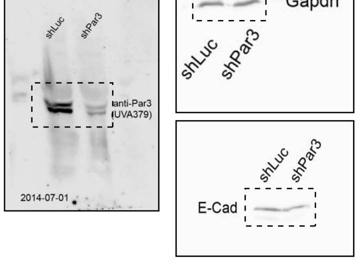

Figure 4f

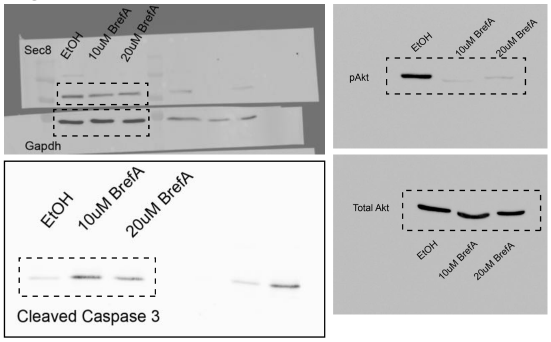

Figure 6c

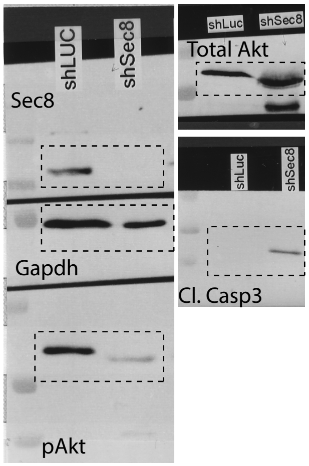

Figure 6d

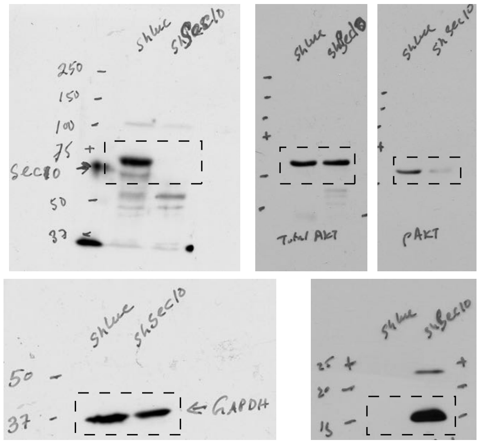

Figure 6e

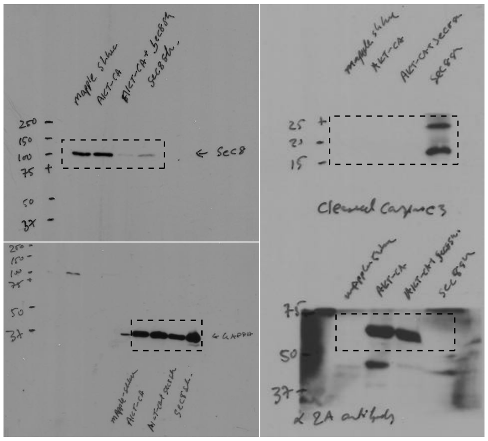

Figure 7a

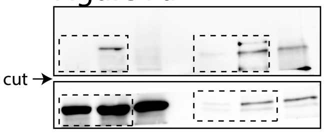

Figure 7b

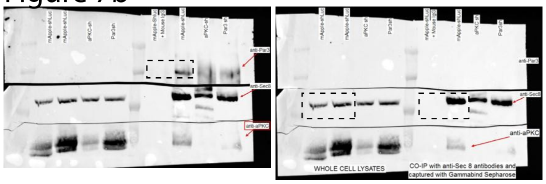

Figure 7g

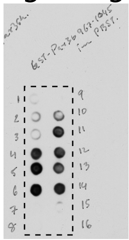

Figure 7i

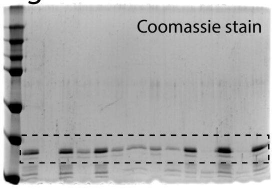

Figure 7k

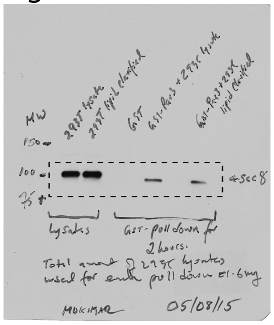

Figure 7l

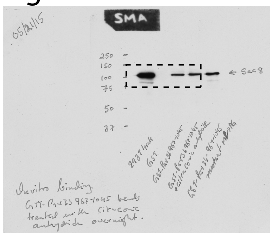

Figure 7m

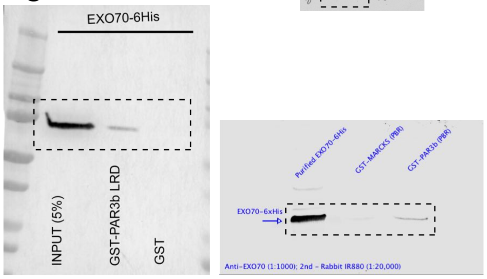

Figure 9f

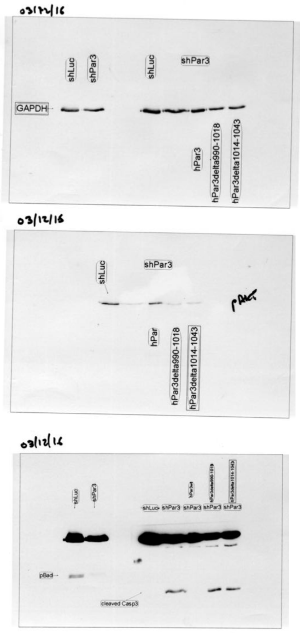

Figure 8b

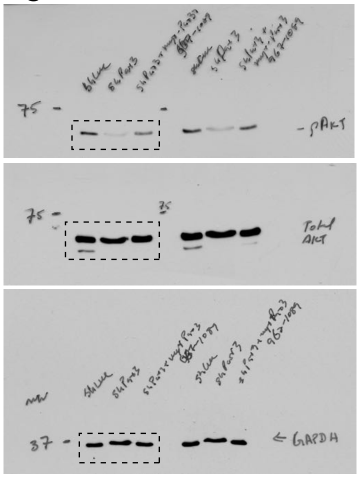

Figure 8d

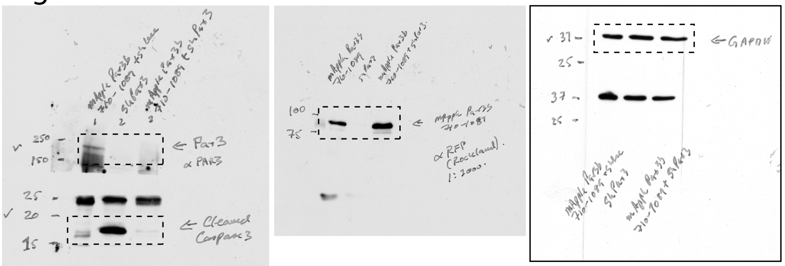

Figure 9a

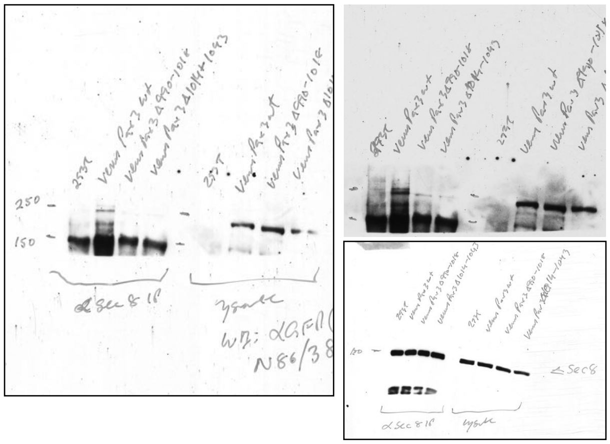

Figure 9c

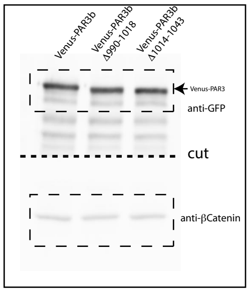

Supplmentary Figure 2d

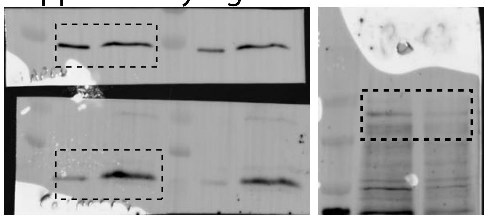

Supplementary Figure 4b

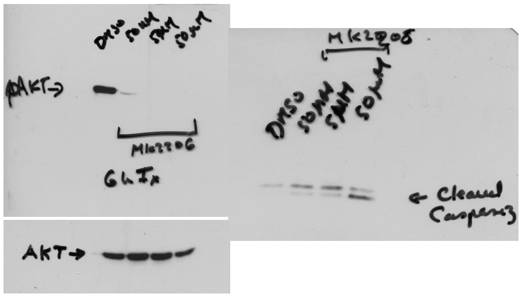

Supplementary Figure 4e

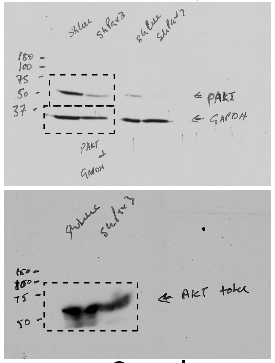

Supplementary Figure 4f

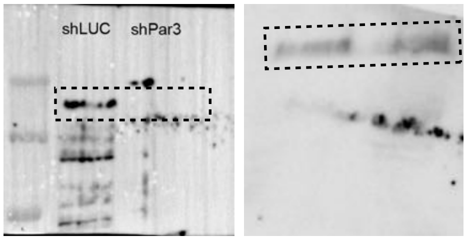

Supplementary Figure 4g

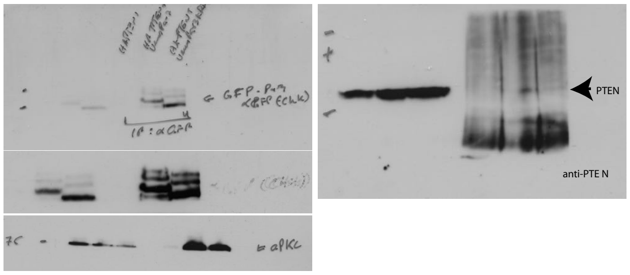

Supplementary Figure 6a

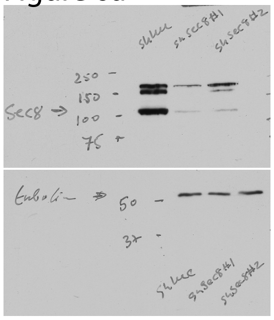

Supplementary Figure 7a

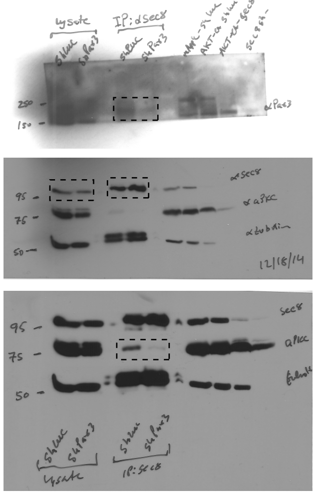

Supplementary Figure 7b

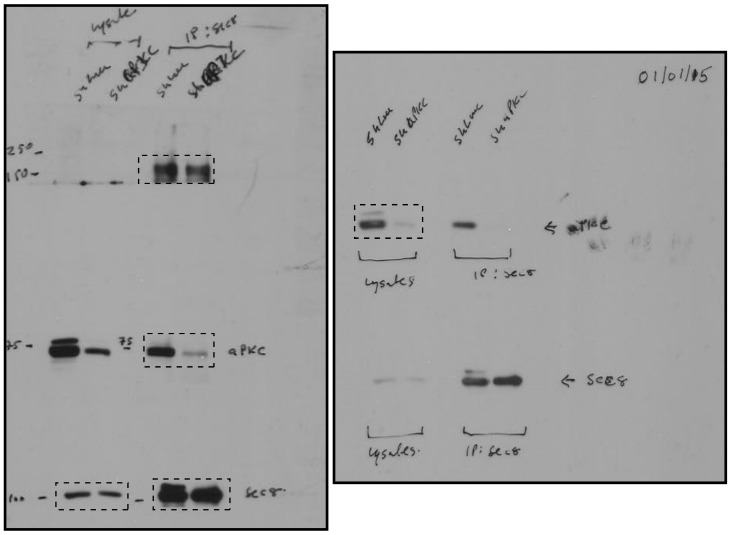

Supplementary Figure 7c

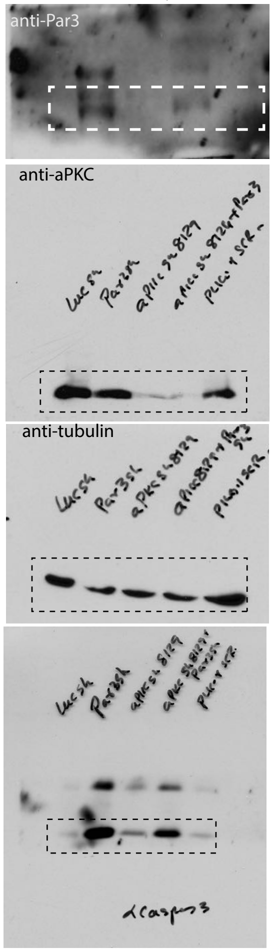

Supplementary Figure 7d

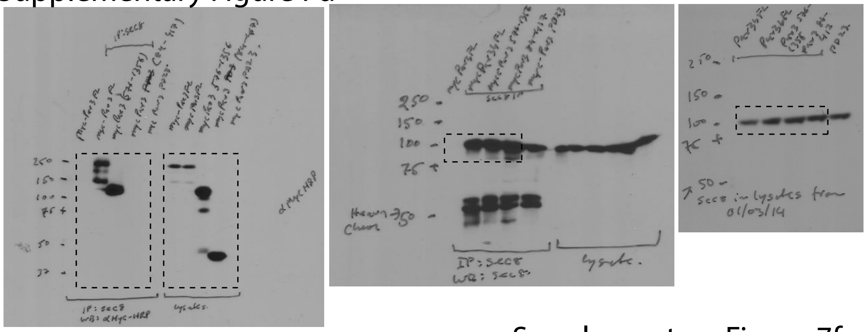

Supplementary Figure 7e

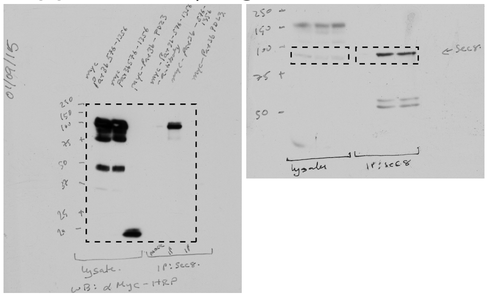

Supplementary Figure 7f

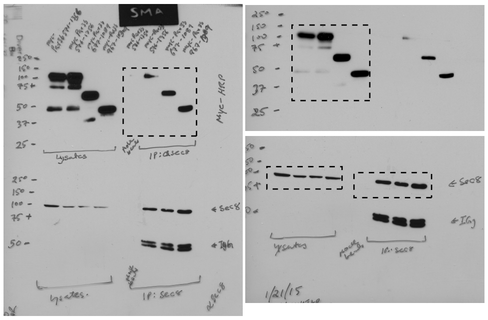

Supplementary Figure 8. Raw western blot scans for all the western blot images shown in this paper.
